# Function of pea amino acid permease AAP6 in nodule nitrogen metabolism and export, and plant nutrition

**DOI:** 10.1093/jxb/ery289

**Published:** 2018-08-03

**Authors:** Matthew G Garneau, Qiumin Tan, Mechthild Tegeder

**Affiliations:** School of Biological Sciences, Washington State University, Pullman, WA, USA

**Keywords:** Amino acid transport, legume, nitrogen fixation, nodule metabolism, nodule nitrogen export, shoot and root nutrition

## Abstract

Legumes fix atmospheric nitrogen through a symbiotic relationship with bacteroids in root nodules. Following fixation in pea (*Pisum sativum* L.) nodules, nitrogen is reduced to amino acids that are exported via the nodule xylem to the shoot, and in the phloem to roots in support of growth. However, the mechanisms involved in amino acid movement towards the nodule vasculature, and their importance for nodule function and plant nutrition, were unknown. We found that in pea nodules the apoplasmic pathway is an essential route for amino acid partitioning from infected cells to the vascular bundles, and that amino acid permease PsAAP6 is a key player in nitrogen retrieval from the apoplasm into inner cortex cells for nodule export. Using an miRNA interference (miR) approach, it was demonstrated that PsAAP6 function in nodules, and probably in roots, and affects both shoot and root nitrogen supply, which were strongly decreased in *PsAAP6*-miR plants. Further, reduced transporter function resulted in increased nodule levels of ammonium, asparagine, and other amino acids. Surprisingly, nitrogen fixation and nodule metabolism were up-regulated in *PsAAP6*-miR plants, indicating that under shoot nitrogen deficiency, or when plant nitrogen demand is high, systemic signaling leads to an increase in nodule activity, independent of the nodule nitrogen status.

## Teaser

Decreased amino acid transporter function in pea nodules affects plant nitrogen status with positive consequences for atmospheric nitrogen fixation and nodule function

## Introduction

Leguminous plant species are able to access atmospheric di-nitrogen (N_2_) through a symbiotic relationship with *Rhizobium* bacteria that are housed in unique root structures called nodules. Bacteroids, a differentiated form of rhizobia, reduce N_2_ within symbiosomes to ammonia (Bond, 1948; Brewin, 1991; White *et al.*, 2007).  The resulting ammonia is transported across the symbiosome membrane into the infected nodule cells and assimilated to glutamine, asparagine, and other amino acids (Atkins *et al.*, 1982; White *et al.*, 2007). Asparagine functions as the dominant N transport form in temperate legumes such as pea (*Pisum sativum*), fava bean (*Vicia faba*), and alfalfa (*Medicago sativa*), while, in tropical legumes such as soybean (*Glycine max*) and common bean (*Phaseolus vulgaris*), ureides are produced and serve as primarily N transport compounds (Layzell *et al.*, 1981; Atkins and Smith, 2007; Tegeder, 2014). Following synthesis, asparagine, other amino acids, or ureides are exported from the nodule via the xylem to the shoot, or in the phloem to the root for metabolism and growth (Pate *et al.*, 1969, 1979; Atkins *et al.*, 1982; Tegeder, 2014).

**Fig. 1.**
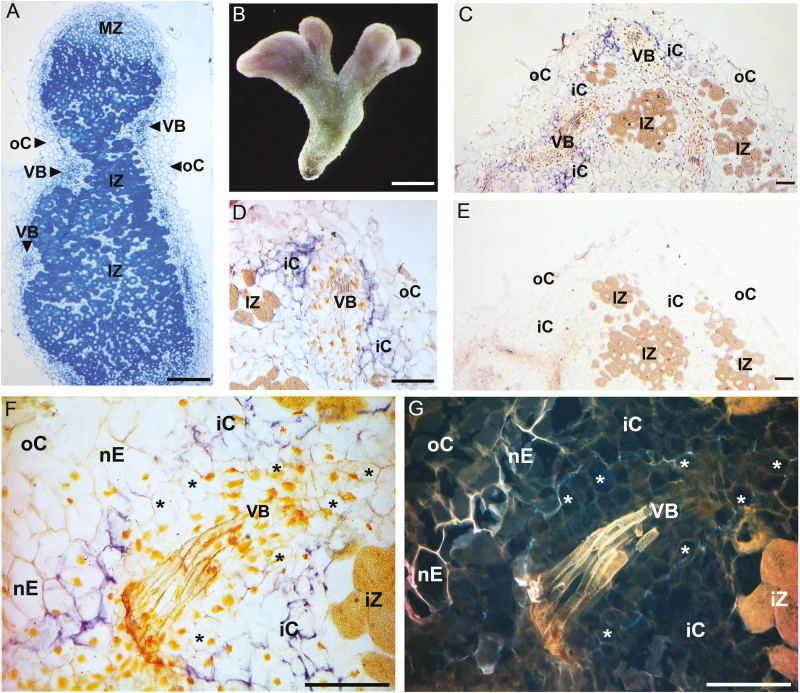
*PsAAP6* localization in pea nodules using *in situ* RNA hybridization. (A) Cross-section of resin-embedded wild-type nodules stained with 1% toluidine blue. (B) Morphology of a wild-type pea nodule. (C–G) Nodule paraffin sections treated with digoxigenin (DIG)-labeled *PsAAP6* antisense (C–D, F) and sense (E) riboprobes. (C, D, F) *PsAAP6* expression is indicated by purple staining and is restricted to the inner cortex cells. (G) UV image of (F) visualizing the lignin in the Casparian strip and the suberin lamellae (white autofluorescence) of the vascular (asterisk) and nodule endodermis (nE). *PsAAP6* is expressed in the inner cortex cells but not in the vascular endodermis. Note that the vascular endodermis shows asymmetric deposition of the Casparian strip toward the inner cortex cells, in contrast to a symmetric arrangement of the apoplasmic barrier in the nodule endodermis (see Hartmann *et al.*, 2002). Yellowish spots in (D) and (F) most probably show nuclei as a result of the preparative procedure. iC, inner cortex cell; IZ, infected zone; MZ, meristematic zone; nE, nodule endodermis; oC, outer cortex cell; VB, vascular bundle. Scale bars=250 µm (A), (B) 2 mm (B), and 100 µm (C–G).

**Fig. 2.**
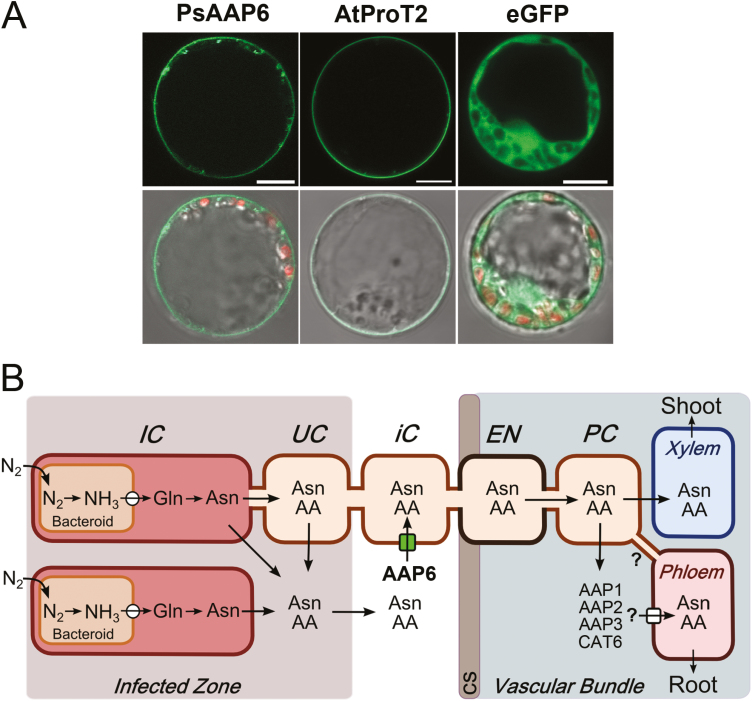
Membrane localization of PsAAP6 and model of PsAAP6 function in pea nodules. (A) Subcellular localization of PsAAP6. PsAAP6–GFP fusion proteins were transiently expressed in pea protoplast (left column). *Arabidopsis thaliana* proline transporter AtProT2 (Grallath *et al.*, 2005) fused to GFP was used as a control for plasma membrane localization (middle column), and free GFP for expression in the cytoplasm (right column). Top row: detection of green fluorescence using confocal laser-scanning microscopy. Bottom row: overlay of confocal images showing GFP fluorescence and chloroplast autoflorescence (red) with corresponding bright-field images. Scale bars=10 µm. (B) Model of nitrogen (N) fixation, N assimilation, and amino acid synthesis in pea nodules. Amino acids, and especially asparagine, are the main N compounds transported out of the nodules in the xylem to the shoot, and in the phloem to the root. The function of PsAAP6 in import of amino acids into the inner cortex cells is indicated by an arrow with a green square. An arrow with a white circle indicates transporter(s) for ammonia (NH_3_) in the symbiosome membrane, and with a white square amino acid transporter(s) functioning in nodule phloem loading. Question marks refer to potential symplasmic connections (i.e. plasmodesmata) between the phloem and surrounding cells, as well as to proposed transporters involved in amino acid phloem loading. AA, amino acids; Asn, asparagine; Gln, glutamine; CS, Casparian strip; EN, endodermis; IC, infected cell; iC, inner cortex cell; PC, pericycle/parenchyma cell; UC, uninfected cell.

Temperate legumes, including pea, develop indeterminate nodules in which the uninfected meristem continues to differentiate, forming a cylindrical or coralloid structure (Brewin, 1991; Fig. 1A, B). The central N-fixing zone contains both infected and uninfected cells, and is surrounded by the inner cortex, peripheral vasculature, nodule endodermis, and outer cortex (cf. Figs 1 and 2B; Bond, 1948; Bergersen, 1982). Each vascular bundle is encircled by an endodermis with a Casparian band that blocks apoplasmic flow of amino acids from the inner cortex cells to the xylem and phloem (Hartmann *et al.*, 2002). Pericycle cells separate the vascular endodermis from the vascular tissue, which consists of parenchyma, phloem sieve element and companion cells, and xylem vessels (see Fig. 1F, G). In indeterminate nodules, plasmodesmata are present between infected and uninfected cells of the N-fixing zone, as well as between other nodule cells (Abd-Alla *et al.*, 2000; Schubert, 2007). These connections would generally allow symplasmic movement of N metabolites between the infected cells and the vasculature. However, the frequency of plasmodesmata between infected cells can be small, and not every infected cell is connected to an uninfected cell since the number of uninfected cells in the central zone can be low (Brown and Walsh, 1994; Schubert, 2007). Together, this suggests that, following synthesis, asparagine and other amino acids must be released into the apoplasm and move in the cell wall space towards the vasculature (see Pate *et al.*, 1969; Peiter *et al.*, 2004). However, to circumvent the apoplasmic blockage of the Casparian band for subsequent transport out of the nodules, reimport of amino acids into the symplasm needs to occur at the latest when the organic N reaches the inner cortex or endodermal cells (see Figs 1F, G, 2B). This import step would require the function of plasma membrane-localized amino acid transporters.

Over the last decades, molecular studies on nodule transport processes have mainly focused on N transfer across the symbiosome membrane into the host cytoplasm (for reviews, see White *et al.*, 2007; Udvardi and Poole, 2013; Clarke *et al.*, 2014). While recent work in soybean demonstrated the role of UPS1 (ureide permease) in ureide transport out of the nodule (Collier and Tegeder, 2012; Carter and Tegeder, 2016), the mechanism of amino acid export from nodules of temperate legumes has not been resolved. Further, it was unknown if transporter function downstream of the infected cells is required for nodule to shoot N partitioning. Amino acid transporters have mainly been characterized in Arabidopsis, and specifically amino acid permeases (AAPs) were shown to function in long-distance transport and source to sink partitioning of the organic N (Fischer *et al.*, 2002; Tegeder and Rentsch, 2010; Tegeder and Masclaux-Daubresse, 2018). AAPs are localized to the plasma membrane and involved in cellular import of a broad spectrum of amino acids. Transcriptome data from *Medicago* resolved that homologs of the Arabidopsis transporters are also expressed in nodules (Benedito *et al.*, 2010; Limpens *et al.*, 2013), potentially facilitating amino acid flow out of the nodules.

The current study supports that in pea nodules the apoplasmic pathway is critical for amino acid movement towards the vascular bundles and requires the function of PsAAP6 to pass the apoplasmic blockage of the vascular endodermis. RNA localization studies showed that *PsAAP6* is expressed in the inner cortex cells of pea nodules, indicating its role in amino acid import from the cell wall space into the nodule symplasm to facilitate N movement to the vasculature for shoot and root N supply. This was confirmed using protein localization studies, as well as molecular and biochemical analyses of composite pea plants with a wild-type shoot and down-regulated expression of *PsAAP6* in nodules and roots. PsAAP6 function in shoot and root N supply was evaluated, as well as how reduced amino acid export from nodules affects biological N fixation and downstream nodule metabolism.

## Materials and methods

### Plant material and growth conditions

Chimeric composite *Pisum sativum* (cultivar Bohatyr) plants with a wild-type shoot and transgenic, nodulated hairy roots were generated using a modified protocol according to Collier *et al.* (2005). In brief, pea seeds were surface-sterilized and planted into standard 10 × 20 inch greenhouse trays (TO plastics, Clearwater, MN, USA) with Sunshine potting mix LC-1 consisting of 70% peat, 20% perlite, and 10% limestone (Sun Gro Horticulture, Bellevue, WA, USA). Plants were grown for 2 weeks in a growth chamber (Conviron, Winnipeg, Canada), in a 14 h photoperiod with light intensity of 400 µmol photons m^–2^ s^–1^ and at 22/18 °C day/night temperatures. Pea plants were then cut just below the first true leaf using a sharp razor blade. The shoots were placed with the cut end submerged in a beaker with a quarter-strength Murashige and Skoog (MS) solution (Murashige and Skoog, 1962) containing *Agrobacterium rhizogenes* strain NCPPB 2659 at an OD_600_ of 0.3 for transformation. The beakers with plants were placed in a sealed chamber and vacuum infiltrated for 30 min. Following infiltration, the plants were incubated on the lab bench top under a plastic dome for 6 h at room temperature in the bacterial solution, and then transferred to 3.5 × 3.5 inch square pots (TO plastics) containing Turface MVP^®^ (Profile Products, Buffalo Grove, IL, USA), with the cut end buried 1 cm below the surface. The plants were covered with a plastic dome and kept at room temperature in the dark for 2 d, followed by exposure to continuous fluorescent light (20–25 µmol photons m^–2^ s^–1^) at room temperature for 21 d until ~10 cm long hairy roots developed. During this period, plants were watered daily with deionized water. On day 14, the roots of each plant were inoculated with 8 ml of a modified Hoagland solution without N as described below and containing *Rhizobium leguminosarum* biovar *viciae* strain C1204. The rhizobia were previously cultured for 3 d on solid YMB medium (1 g l^–1^ yeast extract, 10 g l^–1^ mannitol, 0.5 g l^–1^ K_2_HPO_4_, 0.1 g l^–1^ NaCl, 0.2 g l^–1^ MgSO_4_·7H_2_O, 15 g l^–1^ agar; Allen and Allen, 1950) at 28 °C, and then in liquid YMB medium for 2 d at 28 °C. After centrifugation at 3200 *g* for 20 min at room temperature, the cells were re-suspended with the modified Hoagland solution to reach an OD_600_ of 0.08.

After 21 d at room temperature under fluorescent light, the pea plants grown in N-free Turface were transferred back to the growth chamber (Conviron, Winnipeg, Canada), and grown for 6 weeks under environmental conditions described above. The plants were fertilized twice a week with 100 ml of an N-free Hoagland’s solution (0.625 mM K_2_SO_4_, 0.5 mM MgSO_4_, 0.25 mM KH_2_PO_4_, 3 mM CaCl_2_, 20 μM Fe-EDTA, 46 μM H_3_BO_3_, 9 μM MnCl_2_, 0.76 μM ZnSO_4_, 0.32 μM CuSO_4_, and 0.12 μM Na_2_MoO_4_). Nodulated roots were collected for N fixation experiments, and nodules, roots, xylem sap, stems, and leaves were harvested, flash-frozen using liquid N_2_, stored at –80 °C, and used for molecular and biochemical analyses. Wild-type nodules were also prepared for *PsAAP6* RNA localization studies.

### *In situ* mRNA hybridization of PsAAP6

A pea amino acid permease (PsAAP; gene accession KX620908.1; protein ID AQY72424.1) was previously identified by functional complementation of a yeast (*Saccharomyces cerevisiae*) transport mutant (Tan, 2010). Due to a 71.8% similarity to Arabidopsis AAP6, a plasma membrane transporter that mediates cellular import of a broad spectrum of amino acids (Fischer *et al.*, 2002; Okumoto *et al.*, 2002; Hunt *et al.*, 2010; Supplementary Fig. S1 at *JXB* online), the pea homolog was named PsAAP6. Organ expression analysis showed that *PsAAP6* transcripts are found throughout the plant including nodules (Supplementary Fig. S2). The localization of *PsAAP6* in pea nodules and roots was determined using *in situ* RNA hybridization. Wild-type pea nodules and root segments were fixed overnight at 4 °C in a solution containing 3.7% formaldehyde (v/v), 5% acetic acid (v/v), and 50% ethanol (v/v), followed by dehydration in a series of 30–100% ethanol and gradual substitution of ethanol with xylene using solutions of 1:2, 1:1, and 2:1 xylene:ethanol, and finally 100% xylene. The nodules and root pieces were then embedded in paraffin (Tegeder *et al.*, 2000), and 10 µm sections were prepared using a rotary microtome (Leica Reichert-Jung, Wetzlar, Germany). Sections were mounted on 3-aminopropylethoxysilane-coated slides (Thermo Fischer Scientific, Waltham, MA, USA) and probed with digoxigenin (DIG)-labeled sense or antisense riboprobes. DIG-labeled probes were produced according to the manufacturer’s instructions (Roche Applied Science, Penzberg, Germany) and by using the non-conserved 5'-untranslated region (UTR) of *PsAAP6* to minimize cross-hybridization with other transporter genes. *In situ* RNA hybridization experiments were performed according to Pélissier *et al.* (2004). Nodule fixation and embedding in London Resin White acrylic (Ted Pella Inc., Redding, CA, USA) as well as sample sectioning and staining with toluidine blue O for structural analysis followed Tan *et al.* (2008). All tissue sections were analyzed with a Leitz Aristoplan phase contrast microscope (Leica Microsystems, Wetzler, Germany). Photographs were taken with a Leica DFC 425 CCD camera and by using Leica LAS software. The endodermis contains the Casparian strip and suberin lamellae (Hartmann *et al*., 2002). Suberin monomers and lignin of the Casparian strip and xylem were visualized using the paraffin-embedded samples from the RNA localization experiments and full-spectrum UV fluorescence excitation coupled with a 400–700 nm emission filter (Leica Microsystems).

### Subcellular localization of PsAAP6 in pea protoplasts

*PsAAP6* cDNA without the stop codon was amplified by PCR and cloned into the *Bam*HI restriction site of the pUC18-*GFP6* (green fluorescent protein 6) vector containing the *Cauliflower mosaic virus* (*CaMV*)-*35S* promoter (Foster *et al.*, 2008). The resulting C-terminal *PsAAP6–GFP6* fusion construct, an *AtProtT2–GFP* control construct for plasma membrane localization (Grallath *et al.*, 2005), and free *GFP6* (Foster *et al.*, 2008) were transiently expressed in etiolated pea protoplasts.

Pea protoplasts were isolated from 7-day-old, dark-grown pea seedlings. Seeds were sterilized, planted on half-strength MS medium (Murashige and Skoog, 1962) containing 1% sucrose, and grown at room temperature. Shoot tips of the etiolated seedlings were collected, dissected into 0.5 mm segments, and protoplasts were isolated and transformed according to Neuhaus and Boevink (2001), but by using an enzyme solution containing 0.6% (w/v) Cellulase Onozuka R10 (Yakult Pharmaceutical Industry, Tokyo, Japan), 0.2% (w/v) Macerozyme R10 (Yakult Pharmaceutical Industry), and 1% (w/v) polyvinylpyrrolidone-40 (PVP-40) (Weichert *et al.*, 2012). Following polyethylene glycol (PEG)-mediated transformation (Neuhaus and Boevink, 2001), pea protoplasts were incubated for 16–20 h at room temperature and in the dark, and then analyzed using a confocal laser-scanning microscope (LSM 510, Zeiss, Oberkochen, Germany).

### Preparation of PsAAP6 artificial miRNA constructs

For down-regulation of *PsAAP6* expression in nodulated pea roots, an artificial miRNA (amiRNA) approach was used (see Fig. 4A; Supplementary Fig. S3). The amiRNA was designed along a non-conserved region of *PsAAP6* for transporter-specific repression by using the Web MicroRNA Designer program (WMD2, http://wmd3.weigelworld.org/). As recommended, the designed amiRNA contained two mismatches when compared with the *PsAAP6* target (Supplementary Fig. S3; Davis *et al.*, 2006; Robertson *et al.*, 2010). When searching the pea genome sequence database (https://www.coolseasonfoodlegume.org/), we found no potential off-target sites. Further, the *PsAAP6*-amiRNA was aligned with other pea amino acid transporters, including amino acid permease genes *PsAAP1*, *2*, and *3*, and the cationic amino acid transporter gene *PsCAT6* analyzed in the current study (Supplementary Fig. S3; see also references in Supplementary Table S1). The differences to *PsAAP* genes and *PsCAT6* were 45–50% at nucleotide sequence levels and 9–10 mismatches, respectively, suggesting little or no likelihood of off-target effects. The *PsAAP6* amiRNA was cloned into the *RS300* vector (Schwab *et al.*, 2006) following Ossowski *et al.* (2008). The construct was cloned into the *Bam*HI/*Hin*dIII site of the *pSU*-*SUin*-*OCSt* shuttle vector containing a super ubiquitin promoter (*pSU*; Perera and Rice, 2002; Collier *et al.*, 2005). The amiRNA complex was then moved into the *Sda*I site of *pBIN19*-*FMVp*-*SUin*-*GUS*-*NOSt* (Collier *et al.*, 2005). The final vector construct was transferred into *Agrobacterium rhizogenes* strain NCPPB 2659 (Veena and Taylor, 2007) and used for production of composite *PsAAP6*-miR plants (see above). An additional construct to control for negative effects of the RNAi machinery was produced using an amiRNA (5'-TACACGCTGAACTTCTGGCGG-3') that targets enhanced *GFP* (*eGFP*; Yang *et al.*, 1996), which is absent from the plant genome.

### Tissue collection and identification of transgenic nodulated roots

Composite plants produce a wild-type shoot, and transgenic and non-transgenic nodulated hairy roots. Each composite plant presents an independent transformation event, and several independent transformation events are obtained per plant given that each transgenic root originates from a single cell (Collier *et al.*, 2005). The number of transgenic nodulated roots that develop on single composite plants is highly variable and may range from <20% to 100% (see Fig. 4C). In the current study, the constructs used for transformation also contained a *UidA* gene encoding β-glucuronidase (GUS; Jefferson *et al.*, 1987) and driven by the constitutive *Figwort mosaic virus* promoter (*pFMV*; Sanger *et al.*, 1990). Introduction of the *UidA* gene allowed identification of transgenic nodules and roots through the histochemical GUS staining procedure (Jefferson *et al.*, 1987; see Fig. 4A, B). At plant harvest, tissues including leaves, stem, xylem sap, each single root, and nodules of the respective root were collected separately from at least 24 plants per construct and stored until transgenic nodulated roots were identified. The individual roots (and their nodules) that developed at the stem base of each pea plant were labeled before storage, and root tip samples were taken for the GUS staining procedure using vacuum infiltration for 15 min at room temperature followed by incubation at 37 °C for 24 h (Tegeder *et al.*, 2007). Once transgenic nodulated roots were identified, nodules and root and shoot materials of composite plants that developed at least 30% transgenic nodulated roots were pooled (see Fig. 4C). Specifically, the leaves, roots, and nodules of four plants were combined to obtain sufficient nodules per pool. At least four independent biological sample pools (*n*≥4) were obtained for analysis.

### RNA expression analyses

Total RNA was isolated from transgenic (+) and non-transgenic (–) *PsAAP6*-miR as well as *GFP*-miR nodules and roots. Organs of at least four pea plants were pooled. RNA extraction was performed using TRIzol reagent (Thermo Fischer Scientific, Waltham, MA, USA) following the manufacturer’s protocol and as originally described by Chomczynski and Sacchi (1987). RNA samples were treated with TURBO DNase (Thermo Fisher Scientific) to eliminate DNA contamination, and cDNA first-strand synthesis was done using MMLV reverse transcriptase (Thermo Fisher Scientific). To determine expression of N transporter and metabolism genes, quantitative real-time reverse–transcription PCR (qRT–PCR) was performed following Zhang *et al.* (2010) and by using a 1:20 cDNA dilution. Primers used were designed along non-conserved DNA regions to ensure gene-specific amplification. For gene accessions, primer information, and references, see Supplemental Table S1. Experiments were performed using an Applied Biosystems 7500 Fast Thermal cycler (Foster City, CA, USA) and with three technical replicates to produce threshold (C_T_) values (Sanders *et al.*, 2009). Fold changes in gene expression were determined by comparing the C_T_ values with the control gene *EF1α* (elongation factor 1 alpha; X96555) using the 2^−ΔΔCT^ method (Livak and Schmittgen, 2001). qRT–PCR experiments were done using nodule RNA from three independently grown sets of plants.

### Analysis of biological nitrogen fixation using stable isotope-labeled ^15^N_2_

Nodulated roots were separated from the shoot of composite *PsAAP6*-miR and *GFP*-miR plants, and labeled. Root tip and nodule samples were taken for the GUS staining procedure (see above) to identify *PsAAP6*-miR (+) and *PsAAP6*-miR (–) nodulated roots. All nodulated roots were placed in a vacuum chamber for N fixation experiments. Following removal of the air, the chamber was filled with a 9% volume of ^15^N_2_ gas. Samples were incubated for 2 h and then transferred to a drying oven set at 70 °C. Nodules exposed to atmospheric gas were used as control. ^15^N-treated and untreated *PsAAP6*-miR (+), *PsAAP6*-miR (–), and *GFP*-RNAi nodules were collected after transgenicity or non-transgenicity was confirmed. The nodules were ground, and sample aliquots (1 mg) were combusted in a Costech ECS 4010 analyzer (Costech, Valencia, CA, USA) and separated by GC. The resulting gas was injected into a Micromass IsoPrime isotope ratio mass spectrometer (IRMS) to determine elemental N as well as ∂^15^N (∂^15^N=1000[R_sample_/R_standard_]–1‰, where R=^15^N/^14^N). Atom % excess ^15^N, reflecting (^15^N) incorporation in N_2_, was calculated and the rate of N fixation was determined as outlined in Warembourg (1993).

### Analysis of nodule ammonium and amino acid concentrations

Nodule ammonium concentrations were determined using a colorimetric-based ammonium assay kit (k-ASNAM; Megazyme, Wicklow, Ireland). In this assay NADPH, 2-oxogluturate, and a bacterial glutamate dehydrogenase (Lund, 1986) were added to the ammonium-containing nodule sample, and NADPH consumption in the formation of glutamate was measured by the decrease in absorbance at 340 nm. Nodules used for analysis were immediately flash-frozen in liquid N_2_ at harvest to avoid deamination or deamidation of amino acids and proteins, and ammonium accumulation (Streeter, 1989), and then freeze-dried. Extracts were obtained from 2 mg of lyophilized tissue and by adding 500 µl of ice-cold deionized water, followed by a centrifugation step (4 °C at 20 000 *g* for 15 min) and filtration of the supernatant through a 0.2 µm nylon micro-spin filter (Thermo Fisher Scientific). The resulting extracts (100 µl per sample) were used in the assay and absorbance was measured using a microplate reader (BioTek synergy HT; Winooski, VT, USA). Absorbance values were compared with a standard curve to calculate sample ammonium concentrations.

Amino acid analysis was performed using extracts from 3 mg of lyophilized nodule, root, stem, and leaf tissues, as well as xylem sap, following Santiago and Tegeder (2016). Xylem sap was obtained from plants with at least 30% transgenic nodulated roots and according to Perchlik and Tegeder (2017). For analysis, xylem sap and nodule extracts were diluted 20-fold, while 10-fold dilutions of stem, leaf, and root extracts were used. Sample derivatization was done with 4-fluoro-7-nitro-2,1,3-benzoxadiazole (Aoyama *et al.*, 2004), and HPLC analysis was performed following Tan *et al.* (2010) using a Waters 2695 separation module with a 2475 multi λ fluorescence detector and Empower2 software (Waters, Milford, MA, USA).

### Statistical analyses

Results are shown for one plant growth set but are representative of results from at least two independently grown sets of plants. Data are generally presented as means ±SD of at least four biological repetitions, with the exception of the qRT–PCR results, which are shown as means of three technical repetitions. Student’s *t*-test was used to determine statistical significance with SigmaPlot 11.0 software (Systat Software, San Jose, CA, USA). Statistical significance is indicated in graphs by asterisks, and was determined by *P*-values less than 0.05 or 0.001 (*P<0.05; **P<0.001).

## Results

### PsAAP6 is targeted to the plasma membrane of nodule inner cortex cells

*In situ* mRNA hybridization was performed to resolve the cellular localization of *PsAAP6* expression in pea nodules (Fig. 1). Results revealed that *PsAAP6* is expressed in the uninfected zone of the indeterminate nodule, which is in line with recent tissue-specific transcriptome studies, including *AAP6* expression analyses, in *Medicago truncatula* nodules (Limpens *et al.*, 2013). *PsAAP6* expression was restricted to the inner cortex cells (Fig. 1C, D), with strongest expression in cortex cells adjacent to the vascular bundle (Fig. 1D, F). The transporter seems not to be expressed in the vascular endodermis. This was further confirmed by high magnification imaging of *PsAAP6* expression (Fig. 1F) and by analysis of the same section using UV light imaging, which allows visualization of the endodermal Casparian strip (i.e. lignin) and suberin lamellae (Fig. 1G). Similar to what has previously been reported for *Vicia faba*, the vascular endodermis in pea nodules showed asymmetric deposition of the Casparian strip (and potentially suberin) toward the inner cortex cells (Fig. 1G; see Hartmann *et al.*, 2002), and *PsAAP6* expression was only observed in the cortex cells surrounding the endodermis (Fig. 1F). Further, *PsAAP6* expression was not found in the nodule phloem or other cells of the vascular cylinder.

PsAAP6–GFP proteins were transiently expressed in pea protoplasts to determine the subcellular localization of the transporter. Using confocal microscopy and Arabidopsis AtProT2 proline transporter as plasma membrane control (Grallath *et al.*, 2005), PsAAP6 was localized to the cell membrane (Fig. 2A). This localization was even more evident when visualizing chloroplasts using autofluorescence (shown in red; Fig. 2A*).* PsAAP6–GFP proteins were detected at the plasma membrane distal to the chloroplasts and cytoplasm. Some fluorescence was also observed in vesicle-like structures, probably due to usage of the constitutive *CaMV-35S* promoter resulting in very high PsAAP6–GFP expression levels and some mistargeting of the fluorescent proteins (Odell *et al.*, 1985). Overall, the protein and RNA localization studies (Figs 1, 2A) support that PsAAP6 is functioning in the inner cortex cells for import of amino acids into the symplasm to promote N movement towards the nodule xylem and phloem (Fig. 2B).

### In roots, PsAAP6 is localized to the phloem and cortex cells

**Fig. 3.**
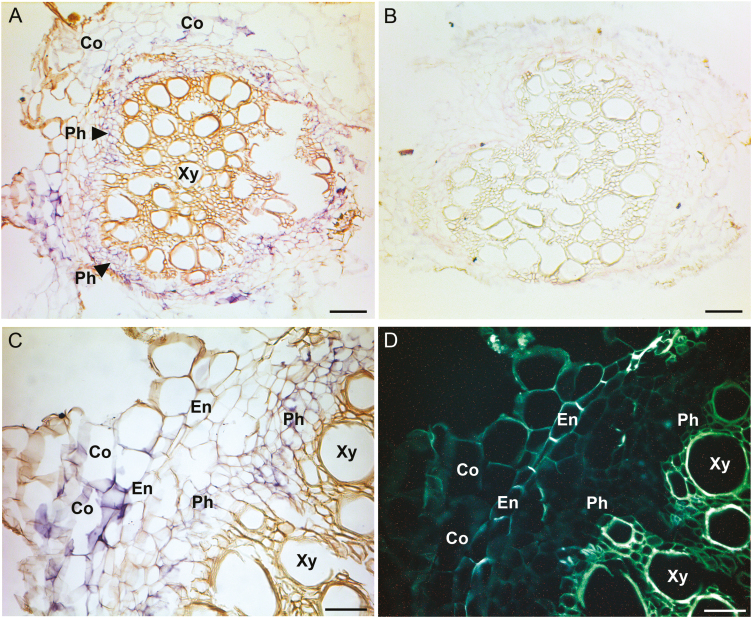
*PsAAP6* localization in pea roots using *in situ* RNA hybridization. (A–C) Root paraffin sections treated with digoxigenin (DIG)-labeled *PsAAP6* antisense (A, C) and sense (B) riboprobes. (A, C) *PsAAP6* expression is indicated by purple staining in both root cortex and phloem cells. (D) UV image of (C) visualizing suberin lamellae and/or lignin (white autofluorescence) of the root endodermis (En) and the xylem vessel elements (Xy). Co, cortex cells; En, endodermis; Ph, phloem; Xy, xylem. Scale bars=100 µm (A, B) and 50 µm (C, D).

Since the approach in the current study was to silence *PsAAP6* in nodulated roots of composite pea plants (see below) and taking into account that *PsAAP6* is also expressed in roots (Supplementary Fig. S2), its cellular localization in roots was determined (Fig. 3). The root xylem is located in the center of the vascular cylinder (i.e. stele), and the phloem is exterior to the xylem (Fig. 3A, C, D). Outside the stele lies the endodermis, which is, like in nodules, the boundary between the cortex and stele*. In situ* mRNA hybridization revealed *PsAAP6* expression in the phloem, including companion, sieve element, and parenchyma cells (Fig. 3A, C, D). In the current study, pea plants were not fertilized with N, and the shoot and roots exclusively received their amino acids from nodules. The majority of nodule-synthesized amino acids are allocated in the xylem to the shoot. However, amino acid transfer from the xylem to the phloem also occurs along the entire long-distance transport path to supply sinks (e.g. roots) directly with N (Pate *et al.*, 1979; Layzell *et al.*, 1981), and some of this amino acid exchange may already take place in the root. Indeed, the localization of *PsAAP6* in the root phloem suggests that the transporter functions in xylem to phloem transfer to facilitate amino acid delivery to roots (Fig. 3A, C).

*PsAAP6* expression was further localized to the root cortex (Fig. 3). Root cortex cells generally have high metabolic activity and require substantial amounts of amino acids to promote root import of apoplastically transported nutrients and water, and to enable accumulation of specialized metabolites for protection against biotic and abiotic stresses (Hassan and Mathesius, 2012; Moussaieff *et al.*, 2013; Tegeder, 2014). In addition, in legume roots, symbiotic bacterial infection occurs in the cortex cells, which requires numerous amino acids for synthesis of protein, enzymes, and metabolites to accommodate the infection and nodulation process. Some of the amino acids delivered to the cortex might leak into the root apoplasm, and expression of *PsAAP6* in the cortex cells suggests a role for the transporter in retrieval of the organic N.

### Silencing of *PsAAP6* in nodulated roots of composite pea plants

**Fig. 4.**
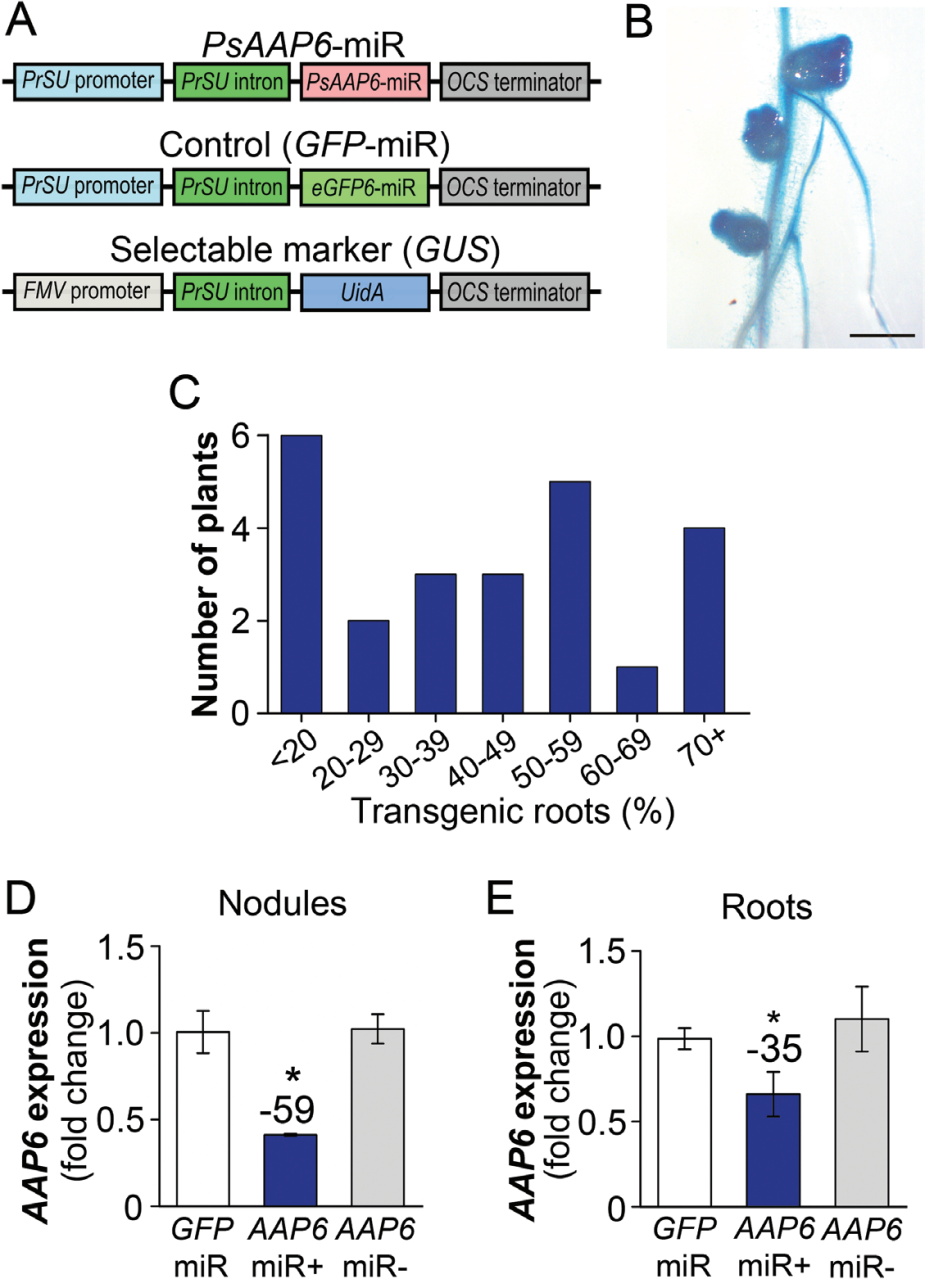
Development of composite pea plants with transgenic *PsAAP6*-miRNA roots. (A) Gene cassettes of *PsAAP6*-miR and control (*GFP*-miR) plasmids used for production of transgenic hairy roots (top and middle). The *UidA* gene encoding β-glucuronidase (GUS) was used as a selectable marker (bottom). For details, see the Materials and methods. (B) GUS-stained transgenic nodulated root. Scale bar=5 mm. (C) Percentage of transgenic roots developing on composite pea plants. (D, E) *PsAAP6* expression analysis in (D) nodules and (E) roots of composite *PsAAP6*-miR and *GFP*-miR pea plants. qRT–PCR experiments were performed with nodule and root RNA from *PsAAP6*-miR and *GFP*-miR (control) plants (*n*=3). Both transgenic (+) and non-transgenic (–) nodules and roots of *PsAAP6*-miR plants were analyzed, as well as transgenic *GFP*-miR nodules and roots. Data are presented as means ±SD. Significant differences are indicated by asterisks (Student’s *t*-test; *P*-values ≤0.05). Numbers above columns describe the percentage change between *PsAAP6*-miR (+) and *GFP*-miR organs.

The production of stable transgenic pea lines is still extremely challenging. Therefore, to be able to analyze PsAAP6 functionally *in planta*, but also to restrict repression of *PsAAP6* expression to nodulated roots, we developed composite pea plants with wild-type shoots and transgenic nodulated roots. This strategy has previously been successfully applied in legumes including pea (Collier *et al.*, 2005; Clemow *et al.*, 2011; Deng *et al.*, 2011). Composite pea plants were produced expressing either an artificial *PsAAP6* miRNA (*PsAAP6*-miR) or, as control, an enhanced GFP (*GFP*-miR) (Fig. 4A). In general, nodules that grow on transgenic roots following infection with *R. leguminosarium* are also transgenic (Fig. 4B). However, since not all nodulated roots that develop on a composite plant contain the miRNA gene cassette, transgenic roots and nodules were identified using the GUS staining procedure (see the Materials and methods). Results showed that the amount of transgenic, nodulated roots varied strongly between individual composite plants (Fig. 4C). Generally, <20% to 100% of the nodulated roots were transgenic, with the majority of plants producing 30–80% transgenic nodulated roots. For further analysis, only composite *PsAAP6*-miR plants that developed at least 30% transgenic nodulated roots were used.

*PsAAP6* expression was analyzed in transgenic (+) and non-transgenic (–) nodules and roots of *PsAAP6*-miR plants, as well as in transgenic *GFP*-miR nodules and roots (Fig. 4D, E). The results demonstrated repression of *PsAAP6* transcripts by ~50% in *PsAAP6*-miR (+) compared with *PsAAP6*-miR (–) and *GFP*-miR nodules (Fig. 4D). Similarly, transporter expression in *PsAAP6*-miR (+) roots was down-regulated by ~35% (Fig. 4E).

### Amino acid delivery from nodule to shoot is decreased in *PsAAP6*-miR plants

**Fig. 5.**
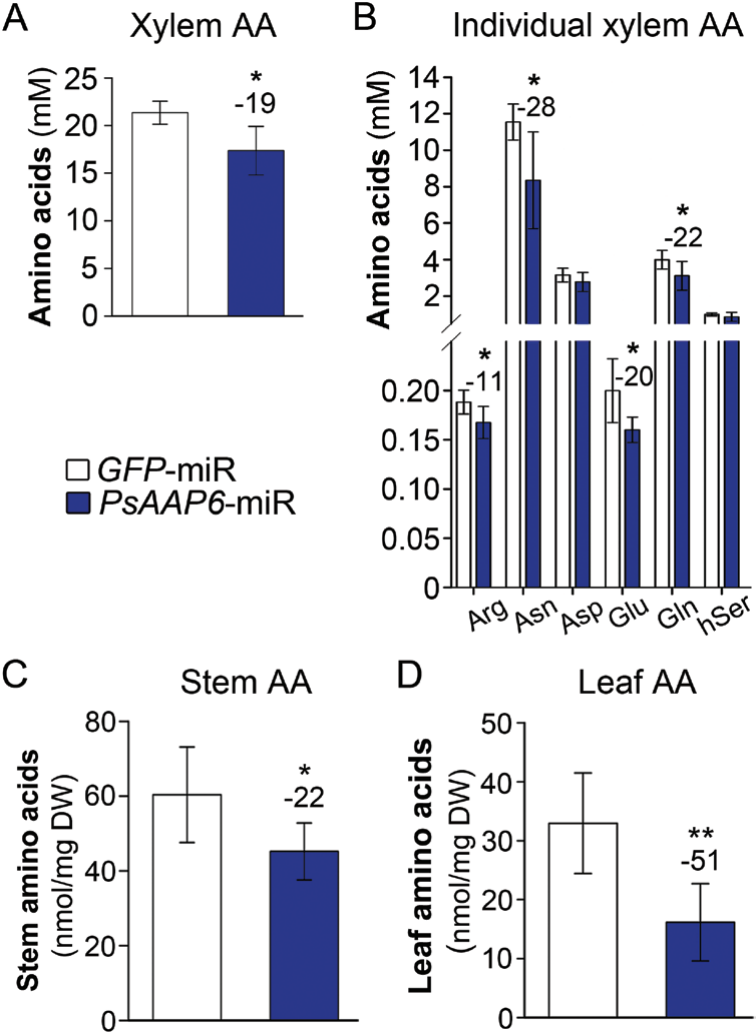
Analysis of xylem and shoot free amino acid levels in *PsAAP6*-miR plants. (A, C, D) Total amino acid (AA) concentrations in (A) xylem (*n*=9), (C) stem (*n*=6), and (D) leaves (*n*=6). (B) Concentrations of selected, individual xylem amino acids. See Supplementary Tables S2 and S3 for full spectrum and concentrations of free amino acids in the xylem, stem, and leaves. Data are presented as means ±SD. Significant differences are indicated by asterisks (Student’s *t*-test; *P<0.05; **P<0.001). Numbers above columns describe the percentage change between *PsAAP6*-miR and *GFP*-miR control plants.

Composite *PsAAP6*-miR plants grown in N-free Turface were not fertilized with N and therefore solely relied on N fixation and amino acid delivery from nodules. Amino acids leave the nodules mainly via the xylem that is connected to the root vasculature. To examine if repression of *PsAAP6* in the nodule inner cortex and in the root affects N transport from nodules to the shoot, xylem amino acid levels were analyzed. As pointed out, composite plants develop both transgenic and non-transgenic roots and nodules (Fig. 4C). Consequently, the collected xylem exudates contained sap and amino acids from both types of nodules. Nevertheless, total xylem amino acid content was reduced by 19% in *PsAAP6*-miR compared with control *GFP*-miR plants (Fig. 5A; Supplementary Table S2), supporting that N translocation from nodules to the shoot decreases when *PsAAP6* expression in nodules is reduced. Since *PsAAP6* is also present in the root phloem, and considering that its expression was down-regulated in *PsAAP6*-miR (+) roots, decreased xylem to phloem transfer and/or re-loading of amino acids leaking out of the root phloem may have further contributed to the observed changes in xylem amino acid levels. Asparagine was most abundant in the xylem, representing ~50% of the total amino acids, and its level was significantly decreased by 28% in *PsAAP6*-miR plants (Fig. 5B; Supplementary Table S2). In addition, amounts of xylem glutamine, glutamate, and arginine were reduced by between 11% and 22%. Decreased nodule to shoot N allocation was further confirmed by analyses of stem and leaf amino acids. Total amino acid levels were reduced in stem and leaves by 22% and 51%, respectively (Fig. 5C, D). In both tissues, this reduction was due to a decrease in a broad spectrum of amino acids (Supplementary Table S3).

### Nodule to root delivery of amino acids is decreased in *PsAAP6*-miR plants

**Fig. 6.**
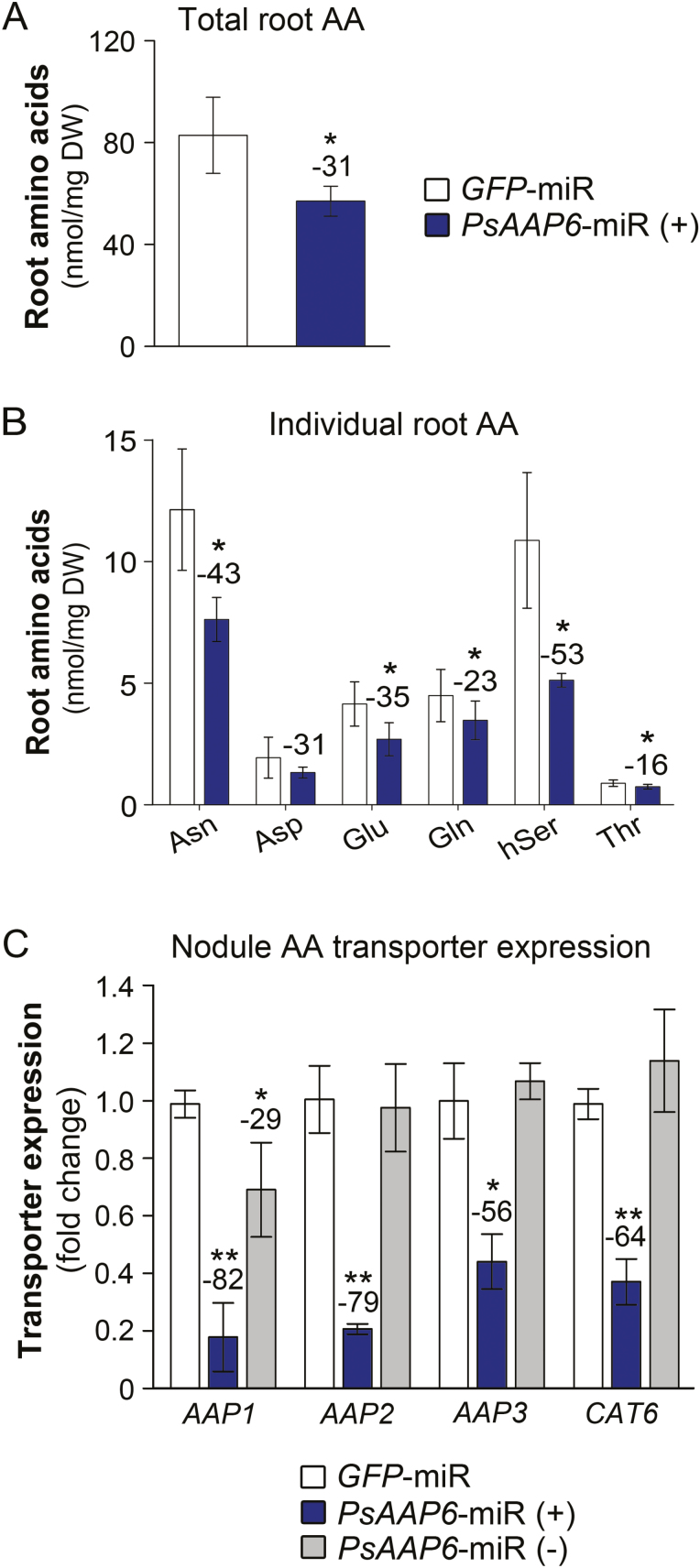
Analysis of amino acid transport from *PsAAP6*-miR nodules to roots. (A) Total free amino acids in transgenic roots of *PsAAP6*-miR and *GFP*-miR plants. (B) Concentrations of selected, individual root amino acids. See Supplementary Table S2 for the full spectrum and concentrations of free root amino acids. (C) Expression analysis of putative amino acid phloem loaders in nodules. Expression levels of pea amino acid permease genes *AAP1*, *AAP2,* and *AAP3*, and the cationic amino acid transporter gene *CAT6* were determined by qRT–PCR using total RNA from transgenic (+) and non-transgenic (–) nodules of *PsAAP6*-miR plants as well as from transgenic *GFP*-miR nodules. See Supplementary Table S1 for gene accessions, primers, and references. Data are presented as means ±SD. Significant differences are indicated by asterisks (*n*≥4; Student’s *t*-test; *P<0.05; **P<0.001). Numbers above columns describe the percentage change between *PsAAP6*-miR (+) and *GFP*-miR roots and nodules.

To analyze if organic N allocation from nodules to root was altered, amino acid levels were measured in the transgenic roots. Results showed a 31% decrease in total amino acid levels in *PsAAP6*-miR (+) versus *GFP*-miR roots (Fig. 6A). Asparagine and homoserine mainly contributed to this reduction, but glutamate, glutamine, and threonine levels were also decreased (Fig. 6B).

In legumes that utilize fixation as the sole source of N, developing roots receive their N either through xylem to phloem transfer (Pate *et al.*, 1979, Layzell *et al.*, 1981) or via direct loading of N into the nodule phloem (Pélissier *et al.*, 2004; Collier and Tegeder, 2012; see Fig. 2B). Transporters that play a role in amino acid phloem loading have been found in Arabidopsis, pea, and other plant species (Okumoto et al., 2002, 2004; Tegeder *et al.*, 2007; Tan *et al.*, 2008; Hunt *et al.*, 2010; Zhang *et al.*, 2010; Santiago and Tegeder, 2016), and transport proteins that might serve phloem-loading function in pea nodules probably include members of the AAP family and CAT6 (Cationic Amino Acid Transporter 6; see Tegeder *et al.*, 2000, 2007; Tan *et al.*, 2010; Zhang *et al.*, 2015; Supplementary Fig. S1). Expression of pea *AAP* and *CAT6* transporters was analyzed in transgenic (+) and non-transgenic (–) nodules of *PsAAP6*-miR plants, as well as in transgenic *GFP*-miR nodules. Results showed that transcript levels of pea *AAP1*, *AAP2*, *AAP3*, and *CAT6* were significantly down-regulated in *PsAAP6*-miR (+) compared with *PsAAP6*-miR (–) and *GFP*-miR nodules (Fig. 6C). Transporter expression was similar in *PsAAP6*-miR (–) and *GFP*-miR nodules, with the exception of a reduced expression of *PsAAP1* in *PsAAP6*-miR (–) nodules. Together, *PsAAP6* localization and expression studies in roots (Figs 3, 4E), and *AAP1*, *AAP2*, *AAP3*, and *CAT6* expression analyses in nodules (Fig. 6C) suggest that both nodule phloem loading and root xylem to phloem transfer are decreased, leading to reduced N supply of *PsAAP6*-miR (+) roots.

### Amino acids and ammonium accumulate in transgenic *PsAAP6*-miR nodules

**Fig. 7.**
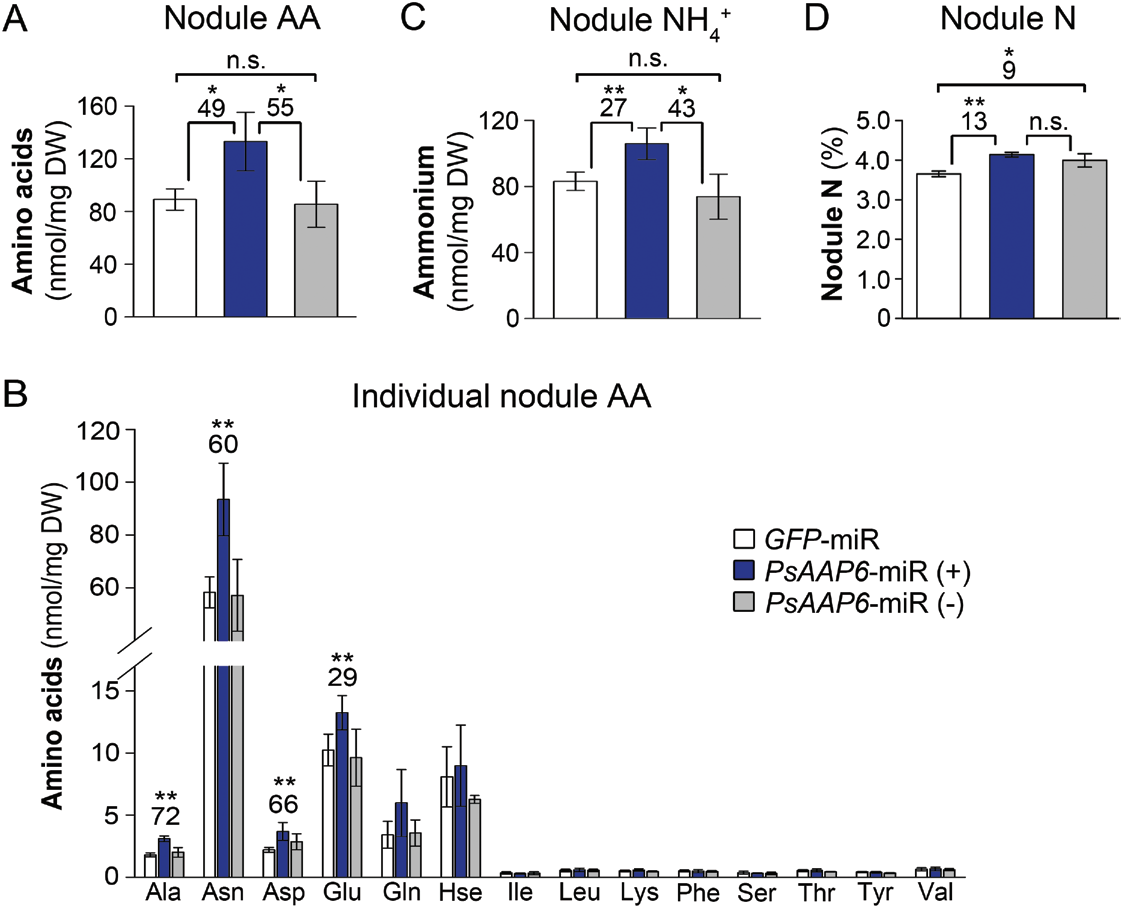
Analysis of nitrogen (N) levels in nodules. Transgenic (+) and non-transgenic (–) nodules of *PsAAP6*-miR plants as well as transgenic *GFP*-miR nodules were analyzed. (A) Total free amino acids (AA). (B) Concentrations and composition of individual amino acids. (C) Total ammonium (NH_4_^+^). (D). Total elemental N. Data are presented as means ±SD. Significant differences are indicated by asterisks (*n*≥4; Student’s *t*-test; *P<0.05; **P<0.001; n.s., not significant). Numbers above columns describe the percentage change between *PsAAP6*-miR and *GFP*-miR control nodules.

We further examined whether and how changes in amino N export from nodules affect nodule N status. HPLC analysis resolved that total amino acid levels in *PsAAP6*-miR (+) nodules were significantly increased by 49% and 55% when compared with *GFP*-miR and *PsAAP6*-miR (–) nodules, respectively (Fig. 7A). Asparagine mainly contributed to this increase, but amounts of other amino acids including alanine, aspartate, and glutamine were also significantly higher (Fig. 7B). Further, ammonium concentrations were increased in *PsAAP6*-miR (+) versus *GFP*-miR or *PsAAP6*-miR (–) nodules (Fig. 7C). No differences in amino acid and ammonium levels were observed between *PsAAP6*-miR (–) and *GFP*-miR nodules. In addition, *PsAAP6*-miR (+) and *PsAAP6*-miR (–) nodules showed an increase in total elemental N by 13% and 9%, respectively, in comparison with *GFP*-miR (Fig. 7D). Overall, the data suggest that reduced amino acid export from *PsAAP6*-miR (+) nodules results in an accumulation of both ammonium and amino acids in nodules.

### Nitrogen fixation and metabolism persist in transgenic *PsAAP6*-miR nodules

**Fig. 8.**
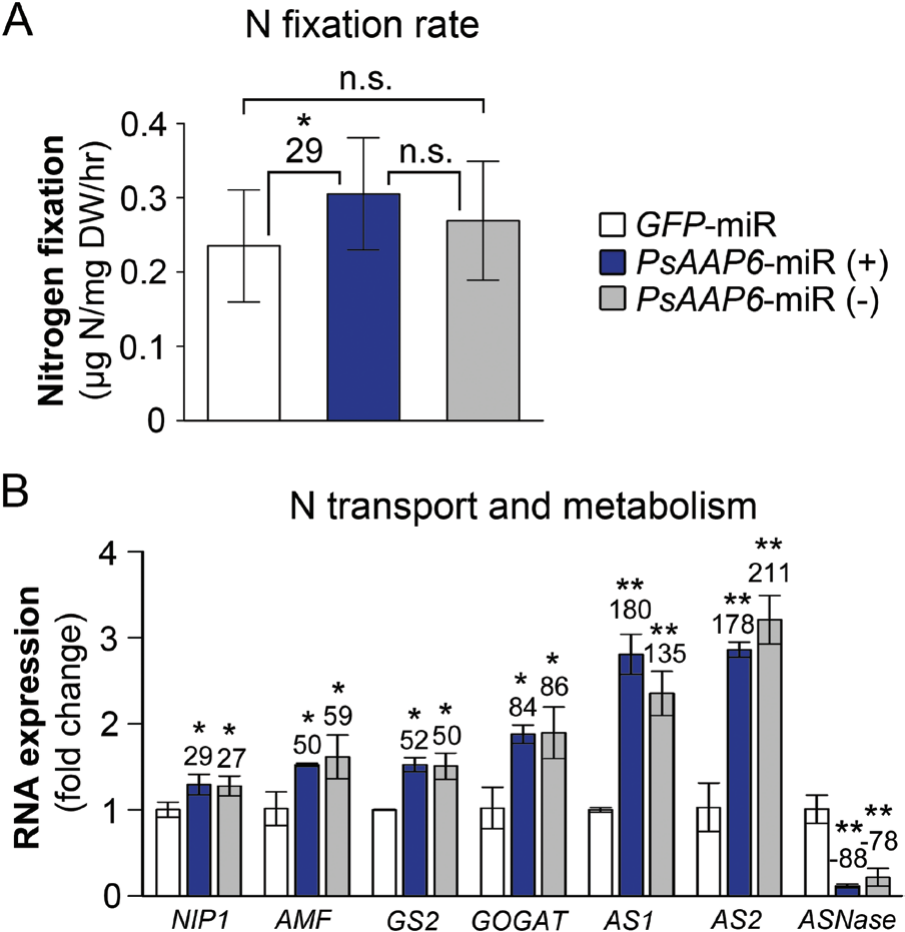
Analysis of nitrogen (N) fixation and assimilation in nodules of *PsAAP6*-miR plants. Transgenic (+) and non-transgenic (–) nodules of *PsAAP6*-miR plants as well as transgenic *GFP*-miR nodules were analyzed. (A) Analysis of N fixation using stable isotope-labeled ^15^N_2_ (*n*≥14; transgenic and non-transgenic nodules from roots of at least 14 plants were analyzed). (B) Expression analysis of N transport and metabolism genes using qRT–PCR and total RNA from *PsAAP6*-miR and *GFP*-miR (control) nodules (*n*=3). Expression of ammonium transporter *NIP1* (*Nodulin 26-like intrinsic protein*) and *AMF* (*ammonium facilitor*) genes, as well as of genes related to N assimilation (*GS2*, *glutamine synthetase*; *GOGAT*, *glutamine 2-oxoglutarate aminotransferase*), asparagine synthesis (*asparagine synthetases AS1*, *AS2*), and asparagine deamination (*ASNase*, *asparaginase*). See Suuplementary Table S1 for gene accessions, primers, and references. Significant differences are indicated by asterisks (Student’s *t*-test; *P<0.05; **P<0.001; n.s., not significant). Numbers above columns describe the percentage change from *GFP*-miR control nodules.

To determine if N acquisition and nodule N metabolism are changed in *PsAAP6*-miR plants, N fixation rates were examined using ^15^N_2_ nodule feeding experiments and subsequent stable isotope analysis of *PsAAP6*-miR and *GFP*-miR nodules. Surprisingly, N fixation was not negatively affected in *PsAAP6*-miR (+) nodules, despite reduced N transport out of nodules, decreased shoot and root N supply, and N accumulation in nodules. On the contrary, N fixation rates were increased by 29% in *PsAAP6*-miR (+) versus *GFP*-miR nodules (Fig. 8A). No differences were found between *PsAAP6*-miR (–) and *PsAAP6*-miR (+) nodules or between *PsAAP6*-miR (–) and *GFP*-miR nodules, but the SDs were relatively high. Finally, it was examined if and how the N fixation rates correlate with ammonium delivery from the bacteroids to the host cell, and with amino acid synthesis in nodules. Expression levels of nodule ammonia/ammonium transporters *NIP1* (*Nodulin 26-like intrinsic protein*) and *AMF* (*ammonium facilitor*), that move ammonium via the symbiosome membrane into the host cell symplasm (Wallace *et al.*, 2006; Chiasson *et al.*, 2014; see Fig. 2B), and of genes related to N assimilation and amino acid synthesis, specifically glutamine synthetase (*GS*2, M20664), 2-oxoglutarate amidotransferase (*GOGAT*, Tan *et al.*, 2010), and asparagine synthase (*AS*, X52179, X52180), were analyzed. In general, transgenic (+) and non-transgenic (–) nodules of *PsAAP6*-miR plants showed similar results (Fig. 8B). Expression of N transport and metabolism genes was significantly up-regulated in both *PsAAP6*-miR (+) and *PsAAP6*-miR (–) compared with *GFP*-miR nodules. On the other hand, *ASNase* encoding asparaginase, that catalyzes the conversion of asparagine to aspartate and ammonia (Tan *et al.*, 2010), was down-regulated in the transgenic and non-transgenic *PsAAP6*-miR nodules (Fig. 8B), suggesting that N is channeled into asparagine pools, either for storage or for export. Together, the data support that despite the differences in amino acid export from transgenic and non-transgenic nodules of *PsAAP6*-miR plants, N fixation and assimilation, and amino acid synthesis are up-regulated in both *PsAAP6*-miR (+) and (–) nodules when compared with *GFP*-miR nodules.

## Discussion

### Transporter function in cortex cells is essential for amino acid export from nodules and controls shoot and root nitrogen supply

In indeterminate pea nodules, fixed N is mainly reduced to asparagine and, to a much lesser extent, other amino acids, which are exported via the xylem or phloem to the shoot and roots, respectively. Two hypotheses have been proposed for movement of N assimilates from the infected zone to the vasculature; amino acids may move symplasmically from cell to cell, and/or they are released into the apoplasm and travel down their concentration gradient towards the inner cortex with its vascular system (Layzell *et al.*, 1981; Peiter *et al.*, 2004; Schubert, 2007; Fig. 2B). However, since the Casparian band of the vascular endodermis blocks further apoplasmic flow to the xylem and phloem (Brown and Walsh, 1994; Hartmann *et al.*, 2002; Doblas *et al.*, 2017), reuptake of amino acids into the symplasm would be required to continue their journey toward the long-distance transport pathways (Abd-alla *et al.*, 2000; Peiter *et al.*, 2004; Schubert, 2007). Localization of PsAAP6 to the plasma membrane and inner cortex cells of pea nodules (Figs 1, 2A) suggests that PsAAP6 is involved in retrieval of amino acids from the apoplasm and facilitates N export out of the nodule (Fig. 2B). This is further supported by analysis of composite *PsAAP6*-miR pea plants showing that repression of *PsAAP6* in nodules results in decreased amino acid delivery to the shoot and root, and in accumulation of amino acids in nodules (Figs 5, 6A, B, 7A, B; Supplementary Tables S2, S3). It is important to point out that the usage of composite plants and an RNAi approach has the advantage that *PsAAP6* expression was only manipulated in nodulated roots. In addition, the composite plants did not receive any N fertilizer and solely relied on fixation of atmospheric N. Therefore, the N/amino acids found in shoot and root tissues can only derive from the nodules, and the observed decreases in amino acid pools in the different *PsAAP6*-miR organs are certainly a result of altered PsAAP6 function in nodules. In addition, down-regulation of *PsAAP6* in the root phloem most probably further affected the amino acid amounts in the *PsAAP6*-miR shoot and roots due to reduced xylem to phloem transfer and reloading of amino acids that leak from the transport phloem (see Fig. 3; Okumoto *et al.*, 2002; Hunt *et al.*, 2010; Zhang *et al.*, 2010).

Alterations were detected for asparagine and other amino acids, indicating that PsAAP6 is mediating transport of a broad spectrum of amino acids, similar to what has been shown for its Arabidopsis homologs (Fischer *et al.*, 2002). Together, these results demonstrate that in indeterminate nodules, the apoplasmic pathway is an essential route for amino acid movement from the infected cells toward the vasculature, and that PsAAP6 is a key player in N uptake from the cell wall space for nodule export. Noticeably, PsAAP6 function in nodule cortex cells seems to affect both downstream xylem and phloem loading of N, since *PsAAP6*-miR plants display reduced amino acid levels in roots, xylem, stem, and leaves (Figs 5, 6A, B).

The reduced *PsAAP6* expression in the nodule inner cortex also led to down-regulation of *CAT6* and other *AAP* transporters in *PsAAP6*-miR (+) nodules (Fig. 6C), probably due to changes in nodule amino acid levels upstream and/or downstream of PsAAP6 function (Hirner *et al.*, 2006; Tan *et al.*, 2010; Zhang *et al.*, 2015; Santiago and Tegeder, 2016). Although the cellular localization of *AAP1*, *AAP2*, *AAP3*, and *CAT6* in nodules still needs to be resolved, based on previous localization or expression studies they may be involved in phloem loading (see Tegeder et al., 2000, 2007; Okumoto *et al.*, 2004; Tan *et al.*, 2010; Zhang *et al.*, 2010, 2015; Tegeder and Ward, 2012; see Fig. 2B) and affect nodule to root amino acid partitioning. Decreased amino acid amounts in *PsAAP6*-miR roots are in line with this assumption (Fig. 6A, B).

This study complements recent work in common bean and soybean plants that produce determinate nodules and use primarily ureides as the long-distance transport form of N. Nodules of both temperate and tropical legumes seem to move their N assimilates apoplasmically towards the vascular bundle and require the activity of AAP and ureide transporters (UPS1), respectively, for nodule N export (this study; Peiter *et al.*, 2004; Collier and Tegeder, 2012; Carter and Tegeder, 2016). However, while UPS1 transporters function in the endodermis as well as the inner cortex cells (Pélissier *et al.*, 2004; Collier *et al.*, 2012), RNA localization studies suggest that PsAAP6 activity is restricted to the cortex cells (Fig. 1). These differences may potentially be due to structural variations in the vascular endodermis dependent on the legume species or the environmental conditions (Guinel, 2009; Barberon, 2017). For example, in contrast to a symmetric arrangement of the Casparian strip in common bean nodules, the vascular endodermis in pea nodules shows an asymmetric deposition of the lignin layer in the cell wall toward the inner cortex cells (Fig. 1F, G; Abd-Alla *et al.*, 2000; Hartmann *et al.*, 2002; Pélissier *et al.*, 2004). Future studies with pea nodules will need to address whether differences in the endodermal structure, including suberization of the transcellular transport pathway (Doblas *et al.*, 2017), hinders uptake of N solutes across the plasma membrane of the endodermal cells, or if as yet unknown transporters mediate amino acid import into these cells.

### Nodule activity is unaffected by the local N status

N fixation and assimilation within the nodule are highly regulated, and previous research in pea has shown that N availability and status are major factors regulating N fixation and downstream assimilation (Jensen, 1987; Schulze, 2004; Fischinger *et al.*, 2006; Sulieman and Tran, 2013). N feedback regulation of N fixation and metabolism may occur (i) indirectly by shoot to nodule signaling or (ii) directly within the nodule through N compounds (Parsons *et al.*, 1993; Schulze, 2004). In nodules, increased levels of ammonium, amino acids, or other N compounds have been associated with negative feedback regulation of nitrogenase and decreased N fixation (Vadez *et al.*, 2000; Serraj *et al.*, 2001; King and Purcell, 2005; Ladrera *et al.*, 2007; Marino *et al.*, 2007; Gil-Quintana *et al.*, 2012; Sulieman and Tran, 2013), and recent work in soybean agrees with this assumption. It showed that decreased ureide transport from nodules and subsequent accumulation of N in nodules negatively affected N fixation and metabolism (Collier and Tegeder, 2012). However, other studies suggest that N fixation occurs independently of ammonium levels and N assimilation (Merrick, 1992; Halbeib and Ludden, 2000; Schulze, 2004). Likewise, an inhibitory effect on N fixation was not observed in *PsAAP6*-miR (+) nodules, although their ammonium, amino acid, and total N levels were strongly elevated (Fig. 7). Whatever the signal, the pea nodule may adjust N partitioning and metabolic processes to avoid inhibition of N fixation and ammonium toxicity, similar to what has been shown for soybean plants with enhanced nodule ureide export (Carter and Tegeder, 2016). Such adjustments may include an ammonium-trapping mechanism by sequestering the N into the apoplasm or vacuole (Britto and Kronzucker, 2002; Simon-Rosin *et al.*, 2003) or increased ammonium flux into the N assimilation pathway, the latter being supported in this study by up-regulation of N assimilation and amino acid synthesis genes (Fig. 8B). In addition, the fixed N in *PsAAP6*-miR (+) nodules seems to be channeled into asparagine pools, which were increased by ~60% (Fig. 7B). As indicated by reduced expression of the asparaginase gene, decreased deamination of asparagine may have further contributed to the high nodule asparagine levels (Figs 7B, 8B). This is in agreement with work in soybean nodules (King and Purcell, 2005; Carter and Tegeder, 2016) and demonstrates that N fixation occurs despite elevated asparagine amounts. It also suggests that in pea plants, asparagine not only serves as a long-distance N transport compound, but also seems to function in ammonium detoxification and as a transient N storage pool (Bauer *et al.*, 1977; Givan, 1979; Bacanamwo and Harper, 1997; Frechilla *et al.*, 2002). Further, based on the current and other pea work, it seems fair to speculate that down-regulation of nitrogenase activity observed in other studies in the presence of high levels of asparagine (Almeida *et al.*, 2000; Sulieman *et al.*, 2010; Esfahani *et al.*, 2014) may not be directly related to the amide. Negative feedback regulation of N fixation could, for example, be caused by modifications in the nodule amino acid spectrum, or changes in the amino acid to elemental N ratio (Schulze, 2003; Fischinger *et al.*, 2006; Larrainzar *et al.*, 2009; Sulieman *et al.*, 2010; Aranjuelo *et al.*, 2011; Gil-Quintana *et al.* 2013; Sulieman and Tran, 2013).

### Up-regulation of N fixation and metabolism may involve a shoot N deficiency signal

Observed increases in asparagine and other amino acids in *PsAAP6*-miR (+) nodules were due to both decreased N export and continuation of N fixation and assimilation (Figs 4D, 5, 6, 7A, B, 8). In fact, *PsAAP6*-miR (+) compared with *GFP*-miR nodules showed a significant increase in N fixation and a subsequent up-regulation of transporters responsible for ammonium movement across the symbiosome membrane into the host cell symplasm (Fig. 8). In addition, transcript levels of N assimilation genes were elevated, supporting increased synthesis of amino acids. Further, N fixation rates were similar in *PsAAP6*-miR (+) and *PsAAP6*-miR (–) nodules, though *PsAAP6*-miR (–) versus *GFP*-miR nodules also showed no difference in N acquisition due to high SDs. Nevertheless, in *PsAAP6*-miR (–) compared with *GFP*-miR nodules, total N levels as well as expression of ammonium transporter and amino acid synthesis genes were increased (Figs 7D, 8B). Overall, these results suggest that N acquisition and amino acid metabolism are up-regulated not only in transgenic but also in non-transgenic nodules of *PsAAP6*-miR plants. Nodule activity is closely related to the shoot N demand, and regulation of N fixation is generally assumed to involve a whole-plant N feedback mechanism co-ordinating shoot N requirements with nodule function (Parsons *et al.*, 1993; Fischinger *et al.*, 2006; Ruffel *et al.*, 2008; Sulieman and Tran, 2013). This process entails the transfer of a shoot signal (or signals) in the phloem to nodules to induce changes in nitrogenase activity (Schulze, 2004; Ruffel *et al.*, 2008; Sulieman and Tran, 2013; Udvardi and Poole, 2013). Leaf amino acid supply was significantly decreased in *PsAAP6*-miR plants by ~50%, which probably induced a phloem-mobile N deficiency signal, triggering an N fixation response in both transgenic and non-transgenic *PsAAP6*-miR nodules. Several studies have addressed systemic down-regulation of nodule activity under environmental stress conditions, such as drought and phosphate deficiencies (Almeida *et al.*, 2000; Schulze *et al.*, 2006; Arrese-Igor *et al.*, 2011). Shoot signals controlling the decrease in nitrogenase activity seem to include asparagine, glutamine, glutamate, aspartate, proline, ureides, or a combination of some of these compounds (Oti-Boateng and Silbury, 1993; Neo and Layzell, 1997; Vadez *et al.*, 2000; Lima and Sodek, 2003; King and Purcell, 2005; Fischinger *et al.*, 2006; Larrainzar *et al.*, 2009; Sulieman *et al.*, 2010). In contrast, up-regulation of N fixation in relation to shoot signaling has, to our knowledge, generally not been addressed, with the exception of a recent study in *Medicago* suggesting γ-aminobutyric acid (GABA) as a long-distance signal and positive regulator of nodule activity (Sulieman and Schulze, 2010). The composite *PsAAP6*-miR pea plants could present a valuable resource for future studies on long-distance signaling and identification of key factors involved in systemic up-regulation of N fixation. Nevertheless, our data suggest (i) that nodule N export processes indirectly influence nodule activity as they influence the shoot (and root) N status, and (ii) that under shoot N deficiency, or when N demand for plant growth is high, systemic signaling leads to an increase of N fixation, uninfluenced by local accumulation of N compounds.

## Supplementary data

Supplementary data are available at *JXB* online.

Table S1. Primers used for gene expression analysis by quantitative real-time PCR (qRT-PCR).

Table S2. Concentrations and spectrum of free amino acids in the xylem and roots of nodulated *PsAAP6*-miR plants.

Table S3. Concentrations and spectrum of free amino acids in the stem and leaves of nodulated *PsAAP6*-miR plants.

Fig. S1. Comparison of pea and Arabidopsis amino acid transporter cDNA sequences.

Fig. S2. Organ expression analysis of *PsAAP6.*

Fig. S3. Alignment of *PsAAP6* amiRNA sequence with pea *AAP* and *CAT6* sequences.

Supplementary Material
