# Supplementary material for: Function of pea amino acid permease AAP6 in nodule nitrogen metabolism and export, and plant nutrition
Source: J Exp Bot. 2018 Aug 3;69(21):5205–19. doi: 10.1093/jxb/ery289 (PMC6184819; doi:10.1093/jxb/ery289)
Supplement: Supplementary Material [file ery289_suppl_supplementary_materials.pdf]

Supplementary data

Table S1. Primers used for gene expression analysis by quantitative real time polymerase chain reaction (qRT-PCR). List of genes, gene accessions, primers and references.

Gene	Gene accessions of pea or legume homologs	Primer sequences (5'-3')	References
Amino acid permease 1 (AAP1)	AY956395	For: GCTGGAACCATTACTGGAGTAAATGA<br>Rev: GACTCTGACGGTGGTGGTGCTTTTAC	Tegeder *et al*., 2000
Amino acid permease 2 (AAP2)	AY956396	For: AGCAAGCCACGAGGATAAGTATAGGC<br>Rev: CAGTGAGTAAGTTTCCTGGCGATGTG	Tegeder *et al.,* 2000
Amino acid permease 3 (AAP3)	KX620905	For: CGTCACAGATTATTGAACATCAAGCA<br>Rev: GGGTCAAAATCACGGGTTGAAATAGA	Tan *et al.,* 2010
Amino acid permease 6 (AAP6)	KX620908	For: GTTAACAGGAGTGCAAGTTGGGGTGG<br>Rev: ATGATTTGGTTCTCTGGAGGGCTTGA	Dhandapani *et al.,* 2016
Cationic amino acid transporter 6 (CAT6)	Partial clone (cf. Tan 2010)	For: GGTTCGGAGTGTTTTCGGCTGTTACG<br>Rev: TGAAACTTTGGTCCACTCTTTCTTTTGA	Tan, 2010
Asparagine synthetase 1 (AS1)	X52179	For: CTGTCACTGCTAGATACCTTGCTGGT<br>Rev: GAGACATCAGAAACATAGGTGTTGCA	Tsai and Coruzzi, 1990
Asparagine synthetase 2 (AS2)	X52180	For: CCATCACTTCTCGCTACCTAGCAACC<br>Rev: TCGACATGAGAAACATAGGCGTGCTT	Tsai and Coruzzi, 1990
L-Asparaginase (ASNase)	*Glycine max*; AY096000<br>*Arachis hypogaea*; DQ889570<br>*Phaseolus vulgaris*; DQ300284	For: GTYATGGAHAARTCYCCDCATTCCTA<br>Rev: AMCARGATTGYDTTTGCYTCCTTTGC	Tan *et al.,* 2010
Glutamate synthase-NADH dependent (GOGAT)	*Phaseolus vulgaris;* AF314925<br>*Medicago sativa;* L37606	For: CACAGATTGCATWGGAACATCCATTC<br>Rev: CCATTTTCATCTCCCAMAAACCTCTTAG	Tan *et al.,* 2010
Glutamine synthetase 2, chloroplastic (GS2)	M20664	For: GAAAATGGCACCATCAATAGGGTAGAG<br>Rev: AGGGATGCGAAACAGGCTTTGATATG	Tingey *et al.,* 1988
Ammonium facilitator 3 (AMF)	*Glycine max*; Glyma08g06880<br>*Medicago truncatula;* Medtr2g010350<br>*Pisum sativum*; CD860511.1	For: CTCGCCTCTTGTCGGAGTTGTCC<br>Rev: CGGTGGTTGTACTTGTCTCCTCTAC	-
Nodulin26-like intrinsic protein 1 (NIP1)	AJ243308	For: CACCGATAACAGAGCGATTGG<br>Rev: TCCATATTCCTCGATATTCATTGTG	Shuurmans *et al.,* 2003
Elongation factor 1α (EF1α)	X96555	For: CAGTGGGACGTGTTGAAACTGGTGTT<br>Rev: ATCGAACATTGTCTCCTGGAAGAGCC	Cong *et al.,* 2000
Ubiquitin (Ubi)	L81139, L81140, L81141, L81142	For: GGCAAAAATACAGGACAAGGAGGGAA<br>Rev: CGCAAMACHAGGTGRAGAGTRGACTC	Xia and Mahon, 1998

Cong X, Ling S, Zhu Y. 2000. Cloning, sequencing and structural analysis of a pea cDNA encoding EF-1α. Chinese Science Bulletin **45**, 338-343.

Dhandapani P, Song J, Novak O, Jameson PE. 2017. Infection by *Rhodococcus fascians* maintains cotyledons as a sink tissue for the pathogen. Annals of Botany **119**, 841-852.

Kawashima K, Suganuma N, Tamaoki M, Kouchi H. 2001. Two types of pea leghemoglobin genes showing different O_2-binding affinities and distinct patterns of spatial expression in nodules. Plant Physiology **125**, 641-651.

Schuurmans JA, van Dongen JT, Rutjens BP, Boonman A, Pieterse CM, Borstlap AC. 2003. Members of the aquaporin family in the developing pea seed coat include representatives of the PIP, TIP, and NIP subfamilies. Plant Molecular Biology **53**, 655-667.

Tan QM. 2010. Understanding the physiological importance of amino acid transport in legumes. Doctoral dissertation, Washington State University.

Tan QM, Zhang LZ, Grant J, Cooper P, Tegeder M. 2010. Increased phloem transport of S-methylmethionine positively affects sulfur and nitrogen metabolism and seed development in pea plants. Plant Physiology **154**, 1886-1896.

Tegeder M, Offler CE, Frommer WB, Patrick JW. (2000) Amino acid transporters are localized to transfer cells of developing pea seeds. Plant Physiology **122**, 319-326.

Tingey S, Tsai FY, Edwards JW, Walker EL, Coruzzi GM.1988. Chloroplast and cytosolic glutamine synthetase are encoded by homologous nuclear genes which are differentially expressed in vivo. Journal of Biological Chemistry **263**, 9651-9657.

Tsai FY, Coruzzi GM. 1990. Dark-induced and organ-specific expression of two asparagine synthetase genes in *Pisum sativum*. The EMBO Journal **9**, 323-332.

Xia X, Mahon J. 1998. Pea polyubiquitin genes:(I) Structure and genomic organization. Gene **215**, 445-452.

Table S2. Concentrations and spectrum of free amino acids in the xylem and roots of nodulated *PsAAP6*-miR plants. Data are presented as means with standard deviation (± SD). Significant differences are indicated by asterisks (Student's *t*-test; *, p<0.05; **, p<0.001). Values for **%**∆ describe the percentage change between *PsAAP6*-miR and *GFP*-miR control plants.

**Amino acids**	**Xylem** (µM)			**Root** (nmol/mg DW)		
	***GFP*-miR**	***AAP6*-miR**	**%∆**	***GFP*-miR**	***AAP6*-miR**	**%∆**
Ala	38.05 ± 5.14	46.75 ± 10.10		0.60 ± 0.11	0.49 ± 0.08	
Arg	188.24 ± 12.10	167.47 ± 16.37*	-11	1.42 ± 0.25	1.08 ± 0.14*	-24
**Asn**	**11543.22 ± 987.22**	**8352.38 ± 2648.52***	**-28**	**12.13 ± 2.50**	**7.62 ± 0.91***	**-37**
Asp	3153.10 ± 380.06	2773.79 ± 519.33		1.93 ± 0.84	1.32 ± 0.23	
Cys	7.58 ± 1.03	8.37 ± 1.24		ND	ND	
Glu	199.81 ± 32.39	160.12 ± 12.93*	-20	4.14 ± 0.91	2.69 ± 0.68*	-35
Gln	3993.25 ± 508.25	3110.05 ± 787.87*	-22	4.48 ± 1.08	3.47 ± 0.80*	-23
Gly	16.19 ± 4.44	16.30 ± 2.45		0.69 ± 0.08	0.75 ± 0.01	
His	79.35 ± 17.21	60.81 ± 23.37		0.19 ± 0.03	0.19 ± 0.02	
Hse	991.85 ± 81.14	860.34 ± 244.88		10.87 ± 2.79	5.12 ± 0.28*	-53
Ile	37.58 ± 4.57	42.88 ± 8.92		0.20 ± 0.04	0.18 ± 0.03	
Leu	42.19 ± 8.95	48.83 ± 13.64		0.23 ± 0.04	0.22 ± 0.04	
Lys	64.10 ± 15.71	73.80 ± 22.47		0.37 ± 0.08	0.35 ± 0.06	
Met	0.46 ± 0.26	0.55 ± 0.17		0.07 ± 0.01	0.05 ± 0.01*	-17
Phe	26.30 ± 6.47	23.38 ± 4.12		0.29 ± 0.01	0.27 ± 0.01*	-7
Pro	9.69 ± 2.55	10.73 ± 2.85		0.11 ± 0.02	0.11 ± 0.01	
Ser	33.10 ± 10.93	31.95 ± 3.69		0.71 ± 0.14	0.75 ± 0.14	
Thr	71.64 ± 9.04	70.66 ± 21.18		0.88 ± 0.13	0.74 ± 0.10*	-16
Tyr	12.02 ± 2.76	9.12 ± 2.43		0.61 ± 0.40	0.65 ± 0.39	
Val	85.12 ± 16.91	86.98 ± 17.09		0.55 ± 0.07	0.53 ± 0.06	
**Total**	**21323.571 ± 1201.81**	**17327.23 ± 2549.27***	**-19**	**41.42 ± 7.47**	**28.49 ± 2.93***	**-31**

Table S3. Concentrations and spectrum of free amino acids in the stem and leaves of nodulated *PsAAP6*-miR plants. Data are presented as means with standard deviation (± SD). Significant differences are indicated by asterisks (Student's *t*-test; *, p<0.05; **, p<0.001). Values for **%**Δ describe the percentage change between *PsAAP6*-miR and *GFP*-miR control plants.

**Amino acids**	**Stem** (nmol/mg DW)			**Leaf** (nmol/mg DW)		
	***GFP*-miR**	***AAP6*-miR**	**%Δ**	***GFP*-miR**	***AAP6*-miR**	**%Δ**
Ala	3.49 ± 0.87	2.82 ± 0.54		3.35 ± 0.51	1.16 ± 0.4**	-65
Asn	13.15 ± 2.39	12.85 ± 1.85		0.35 ± 0.16	0.19 ± 0.06*	-45
Asp	2.43 ± 0.36	2.21± 0.51		2.66 ± 0.22	1.09 ± 0.36**	-59
Cys	0.02 ± 0.01	0.02 ± 0.01		0.25 ± 0.08	0.93 ± 0.65*	270
Glu	12.1 ± 2.42	9.83 ± 1.64		13.18 ± 2.18	4.99 ± 2.08**	-62
Gln	3.76 ± 1.55	3.18 ± 0.98		0.90 ± 0.42	0.30 ± 0.10**	-66
Gly	0.82 ± 0.37	0.71 ± 0.36		2.84 ± 0.51	1.01 ± 0.47**	-64
His	0.30 ± 0.11	0.05 ± 0.02*	-41	0.15 ± 0.04	0.05 ± 0.02**	-69
Hse	7.73 ± 2.94	3.75 ± 1.09*	-51	1.98 ± 0.66	0.72 ± 0.04*	-63
Ile	0.51 ± 0.15	0.33 ± 0.06*	-35	0.20 ± 0.06	0.05 ± 0.02**	-73
Leu	0.304 ± 0.11	0.20 ± 0.06*	-34	0.19 ± 0.06	0.07 ± 0.04*	-60
Lys	0.47 ± 0.12	0.44 ± 0.08		0.27 ± 0.05	0.15 ± 0.05*	-45
Phe	0.34 ± 0.04	0.26 ± 0.02*	-24	0.73 ± 0.11	0.23 ± 0.10**	-68
Pro	4.36 ± 3.36	0.59 ± 0.43*	-86	1.23 ± 0.47	0.38 ± 0.18*	-70
Ser	2.08 ± 0.79	1.74 ± 0.43		1.22 ± 0.42	0.52 ± 0.15*	-58
Thr	1.69 ± 0.47	1.09 ± 0.27*	-36	1.08 ± 0.25	0.33 ± 0.15**	-69
Tyr	0.03 ± 0.01	0.07 ± 0.05		1.49 ± 0.14	0.68 ± 0.48*	-54
Val	0.90 ± 0.18	0.67 ± 0.20		0.43 ± 0.06	0.15 ± 0.05**	-65
**Total**	**56.15 ± 15.39**	**43.55 ± 6.86***	**-22**	**32.96 ± 8.52**	**16.15 ± 7.30***	**-51**

Fig. S1. Comparison of pea and Arabidopsis amino acid transporter cDNA sequences. For gene accessions see Table S1 and http://aramemnon.uni-koeln.de/.

	PsAAP6	***PsAAP1***	***PsAAP2***	***PsAAP3***	***PsCAT6***	***AtAAP1***	***AtAAP2***	***AtAAP3***	***AtAAP4***	***AtAAP5***	***AtAAP6***	***AtAAP7***	***AtAAP8***
PsAAP6	**100**	60.9	56.4	56.4	43	65.6	59.7	62.2	60.1	60.3	71.8	57.9	64.4
PsAAP1	60.9	**100**	60.4	63.6	43.3	59.2	68.4	68.5	65	66.9	61.8	58.6	58.7
PsAAP2	56.4	60.4	**100**	65.7	39.1	53.8	60.4	61.4	62.6	59.1	56.4	52.5	53.2
PsAAP3	56.4	63.6	65.7	**100**	45	55.8	64.3	61.5	60.7	59.1	57	51.5	54.2
PsCAT6	43	43.3	39.1	45	**100**	44.6	42.4	43.4	38.9	41.8	40.4	41.8	42.1
AtAAP1	65.6	59.2	53.8	55.8	44.6	**100**	59.9	60.7	59.3	59.7	69.7	55.6	74.5
AtAAP2	59.7	68.4	60.4	64.3	42.4	59.9	**100**	68.8	77.5	64.9	59.5	55.7	58.3
AtAAP3	62.2	68.5	61.4	61.5	43.4	60.7	68.8	**100**	68.7	68.9	63	59.8	62.2
AtAAP4	60.1	65	62.6	60.7	38.9	59.3	77.5	68.7	**100**	63.5	60.2	54.6	59.4
AtAAP5	60.3	64.9	59.1	59.1	41.8	59.7	64.9	68.9	63.5	**100**	61.5	55.9	60
AtAAP6	71.8	61.8	56.4	57	40.4	69.7	59.5	63	60.2	61.5	**100**	56.7	66.1
AtAAP7	57.9	58.6	52.5	51.5	41.8	55.6	55.7	59.8	54.6	55.9	56.7	**100**	58.4
AtAAP8	64.4	58.7	53.2	54.2	42.1	74.5	58.3	62.2	59.4	60	66.1	58.4	**100**

Fig. S2. Organ-expression analysis of *PsAAP6.* RT-PCR was performed using total RNA from nodulated pea plants grown in potting soil (Sunshine potting mix LC-1 consisting of 70% peat, 20% perlite and 10% limestone) in the greenhouse. Cot, cotyledon; Lf, leaf; Nd, nodule; PW, pod wall; Rt, root; SC, seed coat; St, stem; *18S*, *18 S* ribosomal RNA.

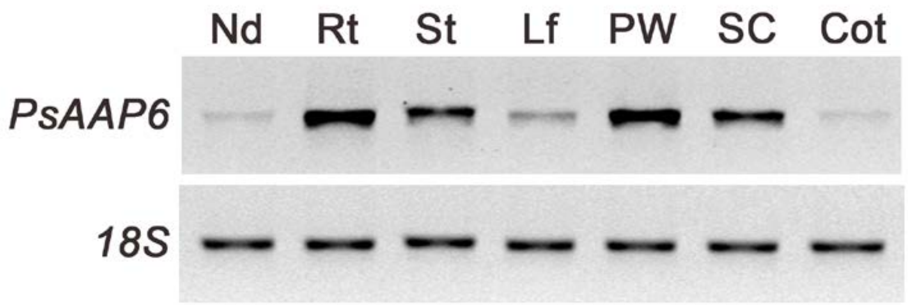

Fig. S3. Alignment of *PsAAP6* amiRNA sequence with pea *AAP* and *CAT6* sequences. Mismatches are marked yellow.

GGCGCGTGCTGTAAGGACAT **- PsAAP6 miRNA 'targeting' coding sequence**
GGAGCGTGCTGTATGGACAT **- PsAAP6 sequence targeted**
20 nucleotides (nt) – 2nt mismatches

GGCG_CGTGCTGTAAGGACAT **- PsAAP6 miRNA 'targeting' coding sequence**
GGATACGCAGCGTTTGGAAAT - **PsAAP1 sequence**
10nt mismatches (50% difference)

GGCGC_GTGCTGTAAGGACAT **- PsAAP6 miRNA 'targeting' coding sequence**
GGAGCAGTTGGGTTTTGGCCT **- PsAAP2 sequence**:
10nt mismatches (50% difference)

GGCGCGTGCTGTAAGGACAT **- PsAAP6 miRNA 'targeting' coding sequence**
GGGGAGTTTTCCAAGGTCTT **- PsAAP3 sequence**
10nt mismatches (50% difference)

GGCGCGTGCTGTA_AGGACAT **- PsAAP6 miRNA 'targeting' coding sequence**:
GGAGAGTTCAGTGGTGAATAT **- PsCAT6 sequence**:
9nt mismatches (45% difference)
